# Supplementary material for: Assembly of recombinant tau into filaments identical to those of Alzheimer’s disease and chronic traumatic encephalopathy
Source: eLife. 2022 Mar 4;11:e76494. doi: 10.7554/eLife.76494 (PMC8983045; doi:10.7554/eLife.76494)
Supplement: Supplementary file 1. [file elife-76494-supp1.docx]

**Cryo-EM data processing, refinement and validation statistics**

**Supplementary table 1**

| **LMB Krios G2** | **2c**  (EMDB-14023)  (PDB 7QJV) |
| --- | --- |
| **Data processing** |  |
| Initial particle images (no.) | 314921 |
| Final particle images (no.) | 37499 |
| Helical twist (º) | -0.797 |
| Helical rise (Å) | 4.75 |
| Symmetry imposed | 1 |
| Map resolution FSC 0.143 (Å) | 3.29 |
| **Refinement** |  |
| Initial model used (PDB code) | 6HRE |
| Model resolution FSC 0.5 (Å) | 3.6 |
| Map sharpening *B* factor (Å^2^) | -22.2 |
| Model composition  Non-hydrogen atoms  Protein residues  Ligands | 6936  912  0 |
| *B* factors (Å^2^)  Protein  Ligand | 49.38  na |
| R.m.s. deviations  Bond lengths (Å)  Bond angles (°) | 0.012  2.254 |
| Validation  MolProbity score  Clashscore  Poor rotamers (%) | 1.23  0.65  0 |
| Ramachandran plot  Favored (%)  Allowed (%)  Disallowed (%) | 90.54  9.47  0 |

**Supplementary table 2**

| **LMB Krios G1** | **4a**  (EMDB-14063)  (PDB 7QL4) |
| --- | --- |
| **Data processing** |  |
| Initial particle images (no.) | 114 023 |
| Final particle images (no.) | 40521 |
| Helical twist (º) | 179.47 |
| Helical rise (Å) | 2.358 |
| Symmetry imposed | 2_1_ |
| Map resolution FSC 0.143 (Å) | 3.20 |
| **Refinement** |  |
| Initial model used (PDB code) | na |
| Model resolution FSC 0.5 (Å) | 3.2 |
| Map sharpening *B* factor (Å^2^) | -62.41 |
| Model composition  Non-hydrogen atoms  Protein residues  Ligands | 3468  456 |
| *B* factors (Å^2^)  Protein  Ligand | 50.01  NA |
| R.m.s. deviations  Bond lengths (Å)  Bond angles (°) | 0.011  1.993 |
| Validation  MolProbity score  Clashscore  Poor rotamers (%) | 1.1  0.28  0 |
| Ramachandran plot  Favored (%)  Allowed (%)  Disallowed (%) | 91.67  8.33  0 |

**Supplementary table 3**

| **LMB Krios G2** | **8a**  (EMDB-14024)  (PDB 7QJW) | **8b**  (EMDB-14062)  (PDB 7QL3) |
| --- | --- | --- |
| **Data processing** |  |  |
| Initial particle images (no.) | 677401 | 677401 |
| Final particle images (no.) | 56911 | 36253 |
| Helical twist (º) | 179.44 | -1.26 |
| Helical rise (Å) | 2.43 | 4.844 |
| Symmetry imposed | 2_1_ | 1 |
| Map resolution FSC 0.143 (Å) | 2.81 | 3.31 |
| **Refinement** |  |  |
| Initial model used (PDB code) | 6NWP | na |
| Model resolution FSC 0.5 (Å) | 2.8 | 3.3 |
| Map sharpening *B* factor (Å^2^) | -108.66 | -95.72 |
| Model composition  Non-hydrogen atoms  Protein residues  Ligands | 3456  450  12 | 3246  426  0 |
| *B* factors (Å^2^)  Protein  Ligand | 115  69.52 | 39.48  na |
| R.m.s. deviations  Bond lengths (Å)  Bond angles (°) | 0.011  2.059 | 0.011  2.067 |
| Validation  MolProbity score  Clashscore  Poor rotamers (%) | 0.74  0  0 | 0.98  0  0 |
| Ramachandran plot  Favored (%)  Allowed (%)  Disallowed (%) | 93.15  4.11  1.37 | 92.27  7.73  0 |

**Supplementary table 4**

| **TFS Krios G4** | **9a**  (EMDB-14026)  (PDB 7QJY) | **9b**  (EMDB-14027)  (PDB 7QJZ) |
| --- | --- | --- |
| **Data processing** |  |  |
| Initial particle images (no.) | 91908 | 91908 |
| Final particle images (no.) | 24754 | 8701 |
| Helical twist (º) | -1.26 | -1.02 |
| Helical rise (Å) | 4.74 | 4.74 |
| Symmetry imposed | 2 | 1 |
| Map resolution FSC 0.143 (Å) | 3.14 | 3.4 |
| **Refinement** |  |  |
| Initial model used (PDB code) | na | na |
| Model resolution FSC 0.5 (Å) | 3.10 | 3.8 |
| Map sharpening *B* factor (Å^2^) | -32.69 | -14.56 |
| Model composition  Non-hydrogen atoms  Protein residues  Ligands | 3444  450  0 | 3378  444  0 |
| *B* factors (Å^2^)  Protein  Ligand | 38.93  na | 39.11  na |
| R.m.s. deviations  Bond lengths (Å)  Bond angles (°) | 0.010  1.965 | 0.011  2.13 |
| Validation  MolProbity score  Clashscore  Poor rotamers (%) | 0.89  0  0.25 | 0.99  0.24  0 |
| Ramachandran plot  Favored (%)  Allowed (%)  Disallowed (%) | 94.29  5.71  0 | 94.44  5.32  0.23 |

**Supplementary table 5**

| **LMB Krios G2** | **10a**  (EMDB-14038)  (PDB 7QK5) | **10b**  (EMD-14320)  (PDB 7R5H) |
| --- | --- | --- |
| **Data processing** |  |  |
| Initial particle images (no.) | 2087042 | 189313 |
| Final particle images (no.) | 108932 | 114483 |
| Helical twist (º) | -1.129 | -0.885 |
| Helical rise (Å) | 4.75 | 4.77 |
| Symmetry imposed | 3 | 1 |
| Map resolution FSC 0.143 (Å) | 1.92 | 2.59 |
| **Refinement** |  |  |
| Initial model used (PDB code) | na | na |
| Model resolution FSC 0.5 (Å) | 1.90 | 2.80 |
| Map sharpening *B* factor (Å^2^) | -13.20 | -37.09 |
| Model composition  Non-hydrogen atoms  Protein residues  Ligands | 5112  666  0 | 3654  477  0 |
| *B* factors (Å^2^)  Protein  Ligand | 49.46  na | 51.37  na |
| R.m.s. deviations  Bond lengths (Å)  Bond angles (°) | 0.011  1.977 | 0.01  1.953 |
| Validation  MolProbity score  Clashscore  Poor rotamers (%) | 0.81  0  0 | 0.92  0.67  0 |
| Ramachandran plot  Favored (%)  Allowed (%)  Disallowed (%) | 95.52  4.48  0 | 96.77  3.23  0 |

**Supplementary table 6**

| **LMB Krios G2** | **11a**  (EMDB-14046)  (PDB 7QKL) |
| --- | --- |
| **Data processing** |  |
| Initial particle images (no.) | 98287 |
| Final particle images (no.) | 74337 |
| Helical twist (º) | 179.402 |
| Helical rise (Å) | 2.378 |
| Symmetry imposed | 2_1_ |
| Map resolution FSC 0.143 (Å) | 2.07 |
| **Refinement** |  |
| Initial model used (PDB code) | na |
| Model resolution FSC 0.5 (Å) | 2.1 |
| Map sharpening *B* factor (Å^2^) | -52.98 |
| Model composition  Non-hydrogen atoms  Protein residues  Ligands | 3456  456  0 |
| *B* factors (Å^2^)  Protein  Ligand | 40.42  na |
| R.m.s. deviations  Bond lengths (Å)  Bond angles (°) | 0.011  2.47 |
| Validation  MolProbity score  Clashscore  Poor rotamers (%) | 0.8  0.14  0 |
| Ramachandran plot  Favored (%)  Allowed (%)  Disallowed (%) | 96.4  3.6  0 |

**Supplementary table 7**

| **LMB Krios G2** | **12a**  (EMDB-14040)  (PDB 7QKF) |
| --- | --- |
| **Data processing** |  |
| Initial particle images (no.) | 51533 |
| Final particle images (no.) | 17783 |
| Helical twist (º) | -1.69 |
| Helical rise (Å) | 4.8 |
| Symmetry imposed | 1 |
| Map resolution FSC 0.143 (Å) | 2.83 |
| **Refinement** |  |
| Initial model used (PDB code) | na |
| Model resolution FSC 0.5 (Å) | 2.2 |
| Map sharpening *B* factor (Å^2^) | -35.91 |
| Model composition  Non-hydrogen atoms  Protein residues  Ligands | 1857  249  0 |
| *B* factors (Å^2^)  Protein  Ligand | 52.51  na |
| R.m.s. deviations  Bond lengths (Å)  Bond angles (°) | 0.011  2.336 |
| Validation  MolProbity score  Clashscore  Poor rotamers (%) | 1.1  0.26  0 |
| Ramachandran plot  Favored (%)  Allowed (%)  Disallowed (%) | 91.56  8.44  0 |

**Supplementary table 8**

| **TFS Krios G4** | **14a**  (EMDB-14053)  (PDB 7QKU) | **14b**  (EMDB-14044)  (PDB 7QKJ) |
| --- | --- | --- |
| **Data processing** |  |  |
| Initial particle images (no.) | 224815 | 224815 |
| Final particle images (no.) | 18013 | 28075 |
| Helical twist (º) | -1.25 | -0.99 |
| Helical rise (Å) | 4.75 | 4.78 |
| Symmetry imposed | 1 | 1 |
| Map resolution FSC 0.143 (Å) | 2.57 | 3.26 |
| **Refinement** |  |  |
| Initial model used (PDB code) | na | na |
| Model resolution FSC 0.5 (Å) | 2.6 | 3.4 |
| Map sharpening *B* factor (Å^2^) | -29.2 | -66.5 |
| Model composition  Non-hydrogen atoms  Protein residues  Ligands | 3456  453  0 | 6888  900  0 |
| *B* factors (Å^2^)  Protein  Ligand | 44.56  na | 38.93  Na |
| R.m.s. deviations  Bond lengths (Å)  Bond angles (°) | 0.01  1.926 | 0.012  2.087 |
| Validation  MolProbity score  Clashscore  Poor rotamers (%) | 0.97  0.28  0 | 1.13  0.21  0 |
| Ramachandran plot  Favored (%)  Allowed (%)  Disallowed (%) | 94.78  5.22  0 | 90.07  9.93  0 |

**Supplementary table 9**

| **LMB Krios G2** | **15a**  (EMDB-14054)  (PDB 7QKV) | **15b**  (EMDB-14056)  (PDB 7QKX) | **15c**  (EMDB-14059)  (PDB 7QL0) |
| --- | --- | --- | --- |
| **Data processing** |  |  |  |
| Initial particle images (no.) | 122945 | 122945 | 122945 |
| Final particle images (no.) | 7127 | 19606 | 14590 |
| Helical twist (º) | -1.3 | 179.426 | -1.33 |
| Helical rise (Å) | 4.75 | 2.37 | 4.75 |
| Symmetry imposed | 3 | 2_1_ | 1 |
| Map resolution FSC 0.143 (Å) | 3.23 | 3.16 | 3.13 |
| **Refinement** |  |  |  |
| Initial model used (PDB code) | na | na | na |
| Model resolution FSC 0.5 (Å) | 3.3 | 3.0 | 3.1 |
| Map sharpening *B* factor (Å^2^) | -54.72 | -45.01 | -46.55 |
| Model composition  Non-hydrogen atoms  Protein residues  Ligands | 4698  621  0 | 3342  438  0 | 3132  414  0 |
| *B* factors (Å^2^)  Protein  Ligand | 101.29  na | 110.93  na | 101.29  na |
| R.m.s. deviations  Bond lengths (Å)  Bond angles (°) | 0.011  1.97 | 0.01  1.88 | 0.01  1.948 |
| Validation  MolProbity score  Clashscore  Poor rotamers (%) | 0.93  0.63  0 | 0.71  0  0 | 0.77  0  0 |
| Ramachandran plot  Favored (%)  Allowed (%)  Disallowed (%) | 96.52  3.48  0 | 96.71  3.29  0 | 96.02  3.98  0 |

**Supplementary table 10**

| **LMB Krios G2** | **16a**  (EMDB-14316)  (PDB 7R4T) |
| --- | --- |
| **Data processing** |  |
| Initial particle images (no.) | 103382 |
| Final particle images (no.) | 32386 |
| Helical twist (º) | 179.483 |
| Helical rise (Å) | 2.39 |
| Symmetry imposed | 2_1_ |
| Map resolution FSC 0.143 (Å) | 2.75 |
| **Refinement** |  |
| Initial model used (PDB code) | na |
| Model resolution FSC 0.5 (Å) | 2.7 |
| Map sharpening *B* factor (Å^2^) | -40.94 |
| Model composition  Non-hydrogen atoms  Protein residues  Ligands | 3468  456  0 |
| *B* factors (Å^2^)  Protein  Ligand | 40.39  na |
| R.m.s. deviations  Bond lengths (Å)  Bond angles (°) | 0.01  1.867 |
| Validation  MolProbity score  Clashscore  Poor rotamers (%) | 0.76  0  0 |
| Ramachandran plot  Favored (%)  Allowed (%)  Disallowed (%) | 96.17  3.83  0 |

**Supplementary table 11**

| **LMB Krios G2** | **20a**  (EMDB-14061)  (PDB 7QL2) |
| --- | --- |
| **Data processing** |  |
| Initial particle images (no.) | 87428 |
| Final particle images (no.) | 55338 |
| Helical twist (º) | -1.62 |
| Helical rise (Å) | 4.78 |
| Symmetry imposed | 1 |
| Map resolution FSC 0.143 (Å) | 2.95 |
| **Refinement** |  |
| Initial model used (PDB code) | na |
| Model resolution FSC 0.5 (Å) | 3.3 |
| Map sharpening *B* factor (Å^2^) | -84.83 |
| Model composition  Non-hydrogen atoms  Protein residues  Ligands | 1107  150  0 |
| *B* factors (Å^2^)  Protein  Ligand | 45.17  na |
| R.m.s. deviations  Bond lengths (Å)  Bond angles (°) | 0.01  2.173 |
| Validation  MolProbity score  Clashscore  Poor rotamers (%) | 0.97  0  0 |
| Ramachandran plot  Favored (%)  Allowed (%)  Disallowed (%) | 92.36  7.64  0 |

**Supplementary table 12**

| **TFS Krios G4** | **23a**  (EMDB-14060)  (PDB 7QL1) |
| --- | --- |
| **Data processing** |  |
| Initial particle images (no.) | 147647 |
| Final particle images (no.) | 23243 |
| Helical twist (º) | -1.25 |
| Helical rise (Å) | 4.75 |
| Symmetry imposed | 1 |
| Map resolution FSC 0.143 (Å) | 3.34 |
| **Refinement** |  |
| Initial model used (PDB code) | na |
| Model resolution FSC 0.5 (Å) | 3.2 |
| Map sharpening *B* factor (Å^2^) | -55.26 |
| Model composition  Non-hydrogen atoms  Protein residues  Ligands | 1107  150  0 |
| *B* factors (Å^2^)  Protein  Ligand | 46.71  na |
| R.m.s. deviations  Bond lengths (Å)  Bond angles (°) | 0.01  2.061 |
| Validation  MolProbity score  Clashscore  Poor rotamers (%) | 1.08  0.29  0 |
| Ramachandran plot  Favored (%)  Allowed (%)  Disallowed (%) | 92.49  7.51  0 |

**Supplementary table 13**

| **TFS Glacios** | **27a**  (EMDB-14058)  (PDB 7QKZ) |
| --- | --- |
| **Data processing** |  |
| Initial particle images (no.) | 1005768 |
| Final particle images (no.) | 54983 |
| Helical twist (º) | -0.75 |
| Helical rise (Å) | 4.70 |
| Symmetry imposed | 3 |
| Map resolution FSC 0.143 (Å) | 2.65 |
| **Refinement** |  |
| Initial model used (PDB code) | na |
| Model resolution FSC 0.5 (Å) | 3.44 |
| Map sharpening *B* factor (Å^2^) | -44.56 |
| Model composition  Non-hydrogen atoms  Protein residues  Ligands | 810  99  0 |
| *B* factors (Å^2^)  Protein  Ligand | 52.47  na |
| R.m.s. deviations  Bond lengths (Å)  Bond angles (°) | 0.012  2.833 |
| Validation  MolProbity score  Clashscore  Poor rotamers (%) | 1.24  0.59  0 |
| Ramachandran plot  Favored (%)  Allowed (%)  Disallowed (%) | 90.12  9.88  0 |

**Supplementary table 14**

| **LMB Krios G2** | **34b**  (EMDB-14025)  (PDB 7QJX) |
| --- | --- |
| **Data processing** |  |
| Initial particle images (no.) | 618020 |
| Final particle images (no.) | 34209 |
| Helical twist (º) | -0.978 |
| Helical rise (Å) | 4.90 |
| Symmetry imposed | 2 |
| Map resolution FSC 0.143 (Å) | 2.99 |
| **Refinement** |  |
| Initial model used (PDB code) | na |
| Model resolution FSC 0.5 (Å) | 3.3 |
| Map sharpening *B* factor (Å^2^) | -63.18 |
| Model composition  Non-hydrogen atoms  Protein residues  Ligands | 6912  912  0 |
| *B* factors (Å^2^)  Protein  Ligand | 40.42  na |
| R.m.s. deviations  Bond lengths (Å)  Bond angles (°) | 0.013  2.517 |
| Validation  MolProbity score  Clashscore  Poor rotamers (%) | 0.56  0.14  0 |
| Ramachandran plot  Favored (%)  Allowed (%)  Disallowed (%) | 98.2  1.69  0.11 |

**Supplementary table 15**

| **TFS Krios G4** | **35d**  (EMDB-14028)  (PDB 7QK1) |
| --- | --- |
| **Data processing** |  |
| Initial particle images (no.) | 214567 |
| Final particle images (no.) | 7045 |
| Helical twist (º) | -1.25 |
| Helical rise (Å) | 4.77 |
| Symmetry imposed | 1 |
| Map resolution FSC 0.143 (Å) | 3.03 |
| **Refinement** |  |
| Initial model used (PDB code) | na |
| Model resolution FSC 0.5 (Å) | 3.1 |
| Map sharpening *B* factor (Å^2^) | -11.08 |
| Model composition  Non-hydrogen atoms  Protein residues  Ligands | 3444  450  0 |
| *B* factors (Å^2^)  Protein  Ligand | 38.93  na |
| R.m.s. deviations  Bond lengths (Å)  Bond angles (°) | 0.01  2.033 |
| Validation  MolProbity score  Clashscore  Poor rotamers (%) | 0.87  0  0 |
| Ramachandran plot  Favored (%)  Allowed (%)  Disallowed (%) | 94.52  5.48  0 |

**Supplementary table 16**

| **TFS Krios G4** | **36a**  (EMDB-14029)  (PDB 7QK2) |
| --- | --- |
| **Data processing** |  |
| Initial particle images (no.) | 564821 |
| Final particle images (no.) | 31627 |
| Helical twist (º) | -1.37 |
| Helical rise (Å) | 4.74 |
| Symmetry imposed | 1 |
| Map resolution FSC 0.143 (Å) | 2.61 |
| **Refinement** |  |
| Initial model used (PDB code) | na |
| Model resolution FSC 0.5 (Å) | 2.53 |
| Map sharpening *B* factor (Å^2^) | -31.51 |
| Model composition  Non-hydrogen atoms  Protein residues  Ligands | 3246  426  0 |
| *B* factors (Å^2^)  Protein  Ligand | 39.48  na |
| R.m.s. deviations  Bond lengths (Å)  Bond angles (°) | 0.01  1.969 |
| Validation  MolProbity score  Clashscore  Poor rotamers (%) | 0.84  0.15  0 |
| Ramachandran plot  Favored (%)  Allowed (%)  Disallowed (%) | 95.89  4.11  0.11 |

**Supplementary table 17**

| **TFS Krios G4** | **38a**  (EMDB-14030)  (PDB 7QK3) |
| --- | --- |
| **Data processing** |  |
| Initial particle images (no.) | 677859 |
| Final particle images (no.) | 37574 |
| Helical twist (º) | -1.49 |
| Helical rise (Å) | 4.74 |
| Symmetry imposed | 1 |
| Map resolution FSC 0.143 (Å) | 2.44 |
| **Refinement** |  |
| Initial model used (PDB code) | na |
| Model resolution FSC 0.5 (Å) | 2.61 |
| Map sharpening *B* factor (Å^2^) | -24.7 |
| Model composition  Non-hydrogen atoms  Protein residues  Ligands | 1335  183  0 |
| *B* factors (Å^2^)  Protein  Ligand | 42.58  na |
| R.m.s. deviations  Bond lengths (Å)  Bond angles (°) | 0.01  2.172 |
| Validation  MolProbity score  Clashscore  Poor rotamers (%) | 0.72  0  0 |
| Ramachandran plot  Favored (%)  Allowed (%)  Disallowed (%) | 96.61  3.39  0 |

**Supplementary table 18**

| **LMB Krios G2** | **39a**  (EMDB-14041)  (PDB 7QKG) |
| --- | --- |
| **Data processing** |  |
| Initial particle images (no.) | 678451 |
| Final particle images (no.) | 165084 |
| Helical twist (º) | -1.18 |
| Helical rise (Å) | 4.79 |
| Symmetry imposed | 1 |
| Map resolution FSC 0.143 (Å) | 3.36 |
| **Refinement** |  |
| Initial model used (PDB code) | na |
| Model resolution FSC 0.5 (Å) | 3.54 |
| Map sharpening *B* factor (Å^2^) | -81 |
| Model composition  Non-hydrogen atoms  Protein residues  Ligands | 1191  162  0 |
| *B* factors (Å^2^)  Protein  Ligand | 44.1  na |
| R.m.s. deviations  Bond lengths (Å)  Bond angles (°) | 0.01  2.054 |
| Validation  MolProbity score  Clashscore  Poor rotamers (%) | 0.92  0  0 |
| Ramachandran plot  Favored (%)  Allowed (%)  Disallowed (%) | 93.59  6.41  0 |

**Supplementary table 19**

| **TFS Krios G4** | **40a**  (EMDB-14039)  (PDB 7QK6) |
| --- | --- |
| **Data processing** |  |
| Initial particle images (no.) | 135479 |
| Final particle images (no.) | 81220 |
| Helical twist (º) | -1.07 |
| Helical rise (Å) | 4.77 |
| Symmetry imposed | 1 |
| Map resolution FSC 0.143 (Å) | 2.27 |
| **Refinement** |  |
| Initial model used (PDB code) | na |
| Model resolution FSC 0.5 (Å) | 2.6 |
| Map sharpening *B* factor (Å^2^) | -27.12 |
| Model composition  Non-hydrogen atoms  Protein residues  Ligands | 1191  162  0 |
| *B* factors (Å^2^)  Protein  Ligand | 44.1  na |
| R.m.s. deviations  Bond lengths (Å)  Bond angles (°) | 0.01  1.958 |
| Validation  MolProbity score  Clashscore  Poor rotamers (%) | 0.85  0  0 |
| Ramachandran plot  Favored (%)  Allowed (%)  Disallowed (%) | 94.87  5.13  0 |

**Supplementary table 20**

| **TFS Krios G4** | **41a**  (EMDB-14042)  (PDB 7QKH) |
| --- | --- |
| **Data processing** |  |
| Initial particle images (no.) | 116487 |
| Final particle images (no.) | 26532 |
| Helical twist (º) | -0.807 |
| Helical rise (Å) | 4.77 |
| Symmetry imposed | 1 |
| Map resolution FSC 0.143 (Å) | 3.17 |
| **Refinement** |  |
| Initial model used (PDB code) | na |
| Model resolution FSC 0.5 (Å) | 3.36 |
| Map sharpening *B* factor (Å^2^) | -9.73 |
| Model composition  Non-hydrogen atoms  Protein residues  Ligands | 1512  204  0 |
| *B* factors (Å^2^)  Protein  Ligand | 33.26  na |
| R.m.s. deviations  Bond lengths (Å)  Bond angles (°) | 0.011  2.473 |
| Validation  MolProbity score  Clashscore  Poor rotamers (%) | 1.51  1.29  0 |
| Ramachandran plot  Favored (%)  Allowed (%)  Disallowed (%) | 85.42  14.58  0 |

**Supplementary table 21**

| **LMB Krios G2** | **42a**  (EMDB-14043)  (PDB 7QKI) |
| --- | --- |
| **Data processing** |  |
| Initial particle images (no.) | 181285 |
| Final particle images (no.) | 82803 |
| Helical twist (º) | 179.59 |
| Helical rise (Å) | 2.38 |
| Symmetry imposed | 2_1_ |
| Map resolution FSC 0.143 (Å) | 3.13 |
| **Refinement** |  |
| Initial model used (PDB code) | na |
| Model resolution FSC 0.5 (Å) | 3.24 |
| Map sharpening *B* factor (Å^2^) | -63.04 |
| Model composition  Non-hydrogen atoms  Protein residues  Ligands | 3468  456  0 |
| *B* factors (Å^2^)  Protein  Ligand | 50.07  na |
| R.m.s. deviations  Bond lengths (Å)  Bond angles (°) | 0.011  2.093 |
| Validation  MolProbity score  Clashscore  Poor rotamers (%) | 1.05  0  0 |
| Ramachandran plot  Favored (%)  Allowed (%)  Disallowed (%) | 89.86  10.14  0 |

**Supplementary table 22**

| **LMB Krios G2** | **43a**  (EMDB-14045)  (PDB 7QKK) |
| --- | --- |
| **Data processing** |  |
| Initial particle images (no.) | 180999 |
| Final particle images (no.) | 84513 |
| Helical twist (º) | -1.29 |
| Helical rise (Å) | 4.77 |
| Symmetry imposed | 1 |
| Map resolution FSC 0.143 (Å) | 2.8 |
| **Refinement** |  |
| Initial model used (PDB code) | na |
| Model resolution FSC 0.5 (Å) | 3.24 |
| Map sharpening *B* factor (Å^2^) | -41.5 |
| Model composition  Non-hydrogen atoms  Protein residues  Ligands | 1734  288  0 |
| *B* factors (Å^2^)  Protein  Ligand | 71.71  na |
| R.m.s. deviations  Bond lengths (Å)  Bond angles (°) | 0.011  2.261 |
| Validation  MolProbity score  Clashscore  Poor rotamers (%) | 1.08  0  0 |
| Ramachandran plot  Favored (%)  Allowed (%)  Disallowed (%) | 88.74  11.26  0 |

**Supplementary table 23**

| **LMB Krios G2** | **44a**  (EMDB-14055)  (PDB 7QKW) |
| --- | --- |
| **Data processing** |  |
| Initial particle images (no.) | 156521 |
| Final particle images (no.) | 9794 |
| Helical twist (º) | -1.01 |
| Helical rise (Å) | 4.78 |
| Symmetry imposed | 1 |
| Map resolution FSC 0.143 (Å) | 2.32 |
| **Refinement** |  |
| Initial model used (PDB code) | na |
| Model resolution FSC 0.5 (Å) | 2.5 |
| Map sharpening *B* factor (Å^2^) | -10.12 |
| Model composition  Non-hydrogen atoms  Protein residues  Ligands | 2268  306  0 |
| *B* factors (Å^2^)  Protein  Ligand | 71.71  na |
| R.m.s. deviations  Bond lengths (Å)  Bond angles (°) | 0.01  2.038 |
| Validation  MolProbity score  Clashscore  Poor rotamers (%) | 0.87  0  0 |
| Ramachandran plot  Favored (%)  Allowed (%)  Disallowed (%) | 94.56  5.44  0 |

**Supplementary table 24**

| **LMB Krios G2** | **45a**  (EMDB-14047)  (PDB 7QKM) |
| --- | --- |
| **Data processing** |  |
| Initial particle images (no.) | 88226 |
| Final particle images (no.) | 29079 |
| Helical twist (º) | 179.59 |
| Helical rise (Å) | 2.4 |
| Symmetry imposed | 2_1_ |
| Map resolution FSC 0.143 (Å) | 2.66 |
| **Refinement** |  |
| Initial model used (PDB code) | na |
| Model resolution FSC 0.5 (Å) | 2.5 |
| Map sharpening *B* factor (Å^2^) | -33.12 |
| Model composition  Non-hydrogen atoms  Protein residues  Ligands | 2436  330  0 |
| *B* factors (Å^2^)  Protein  Ligand | 30.84  na |
| R.m.s. deviations  Bond lengths (Å)  Bond angles (°) | 0.01  1.994 |
| Validation  MolProbity score  Clashscore  Poor rotamers (%) | 0.96  0  0 |
| Ramachandran plot  Favored (%)  Allowed (%)  Disallowed (%) | 92.77  7.23  0 |

**Supplementary table 25**

| **TFS Krios G4** | **47a**  (EMDB-14057)  (PDB 7QKY) |
| --- | --- |
| **Data processing** |  |
| Initial particle images (no.) | 11158705 |
| Final particle images (no.) | 41080 |
| Helical twist (º) | 179.597 |
| Helical rise (Å) | 2.368 |
| Symmetry imposed | 2_1_ |
| Map resolution FSC 0.143 (Å) | 1.86 |
| **Refinement** |  |
| Initial model used (PDB code) | na |
| Model resolution FSC 0.5 (Å) | 2.0 |
| Map sharpening *B* factor (Å^2^) | -21.05 |
| Model composition  Non-hydrogen atoms  Protein residues  Ligands | 3000  402  0 |
| *B* factors (Å^2^)  Protein  Ligand | 30.44  na |
| R.m.s. deviations  Bond lengths (Å)  Bond angles (°) | 0.011  1.967 |
| Validation  MolProbity score  Clashscore  Poor rotamers (%) | 1.02  0.34  0.88 |
| Ramachandran plot  Favored (%)  Allowed (%)  Disallowed (%) | 94.1  5.9  0 |
