# Supplementary figures and images for: Assembly of recombinant tau into filaments identical to those of Alzheimer’s disease and chronic traumatic encephalopathy
Source: eLife. 2022 Mar 4;11:e76494. doi: 10.7554/eLife.76494 (PMC8983045; doi:10.7554/eLife.76494)

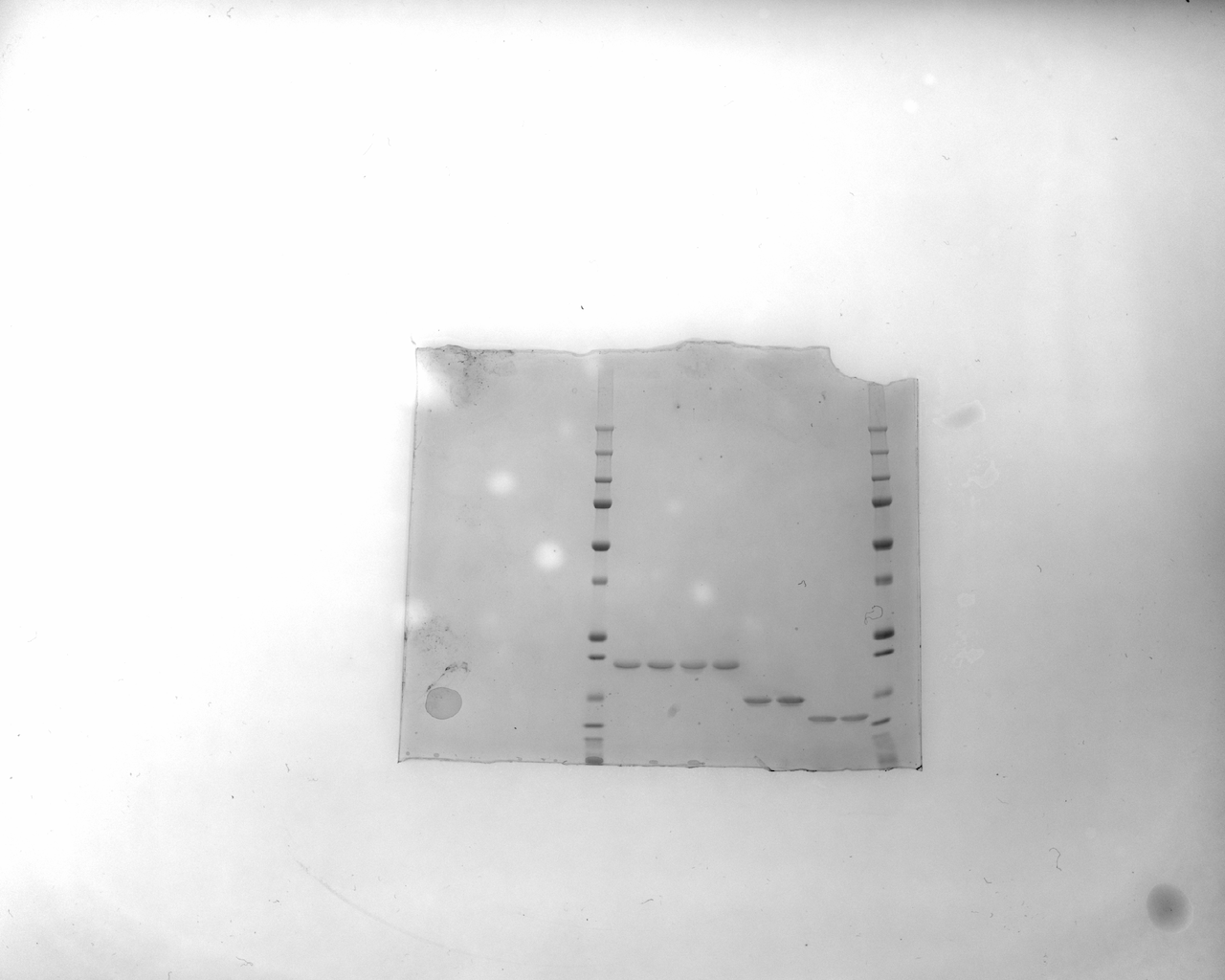

Supplement: Source data 1. [file elife-76494-data1.zip › uncropped_gel.tif]
